# Pharmacokinetic parameters and mechanism of action of an efficient anti-Aβ single chain antibody fragment

**DOI:** 10.1371/journal.pone.0217793

**Published:** 2019-05-31

**Authors:** Gisela Esquerda-Canals, Joaquim Martí-Clúa, Sandra Villegas

**Affiliations:** 1 Protein Design and Immunotherapy Group, Departament de Bioquímica i Biologia Molecular, Unitat de Biociències, Universitat Autònoma de Barcelona, Cerdanyola del Vallès, Spain; 2 Departament de Biologia Cel·lular, Fisiologia i Immunologia, Unitat de Citologia i d’Histologia, Universitat Autònoma de Barcelona, Cerdanyola del Vallès, Spain; Nathan S Kline Institute, UNITED STATES

## Abstract

The success of the targeting of amyloid-β (Aβ) oligomers through immunotherapy in Alzheimer’s disease (AD) mouse models has not been translated into the clinics. The use of single-chain variable fragments (scFvs) has been proposed to prevent the potential severe effects of full-length mAbs by precluding crystallizable fraction-mediated microglia activation. The efficacy of scFv-h3D6, a bapineuzumab-derived anti-Aβ scFv, has been extensively proven. In this work, we compared scFv-h3D6-EL, an elongated variant of the scFv-h3D6, with its original version to assess whether its characteristic higher thermodynamic stability improved its pharmacokinetic parameters. Although scFv-h3D6-EL had a longer half-life than its original version, its absorption from the peritoneal cavity into the systemic compartment was lower than that of the original version. Moreover, we attempted to determine the mechanism underlying the protective effect of scFv-h3D6. We found that scFv-h3D6 showed compartmental distribution and more interestingly crossed the blood–brain barrier. In the brain, scFv-h3D6 was engulfed by glial cells or internalized by Aβ peptide-containing neurons in the early phase post-injection, and was colocalized with the Aβ peptide almost exclusively in glial cells in the late phase post-injection. Aβ peptide levels in the brain decreased simultaneously with an increase in scFv-h3D6 levels. This observation in addition to the increased tumor necrosis factor-α levels in the late phase post-injection suggested that the engulfment of Aβ peptide/scFv-h3D6 complex extruded from large neurons by phagocytic cells was the mechanism underlying Aβ peptide withdrawal. The mechanism of action of scFv-h3D6 demonstrates the effectivity of Aβ-immunotherapy and lays the background for other studies focused on the finding of a treatment for AD.

## Introduction

The currently available therapies for Alzheimer’s disease (AD) include the use of cholinesterase inhibitors (donepezil, galantamine, and rivastigmine) that partially compensate the pathological reduction in acetylcholine and an NMDA receptor antagonist (memantine) that prevents the effect of the increased glutamate levels in the synaptic cleft [1]. Because these treatments palliate the symptoms of AD rather than targeting its underlying causes, the implementation of novel therapeutic strategies has become a necessity [2]. In this sense, several molecules have been designed for targeting amyloid-β (Aβ) peptide, the key component in AD [3,4]. Aβ peptide-directed immunotherapy is a promising approach because it focuses on capturing the Aβ peptide through active immunotherapy (by directing a patient’s immune response to different forms and/or fragments of the peptide) or through passive immunotherapy (by administrating antibodies or their derivatives that directly arrest the Aβ peptide) [5,6]. Passive immunotherapy is a safer option than active immunotherapy because it can be stopped immediately in case of any adverse reaction [7]. Several clinical trials are currently ongoing in this regard [8–10]. Bapineuzumab (Pfizer/Janssen) was the first mAb to reach phase III clinical trials; however, the occurrence of vasogenic edema and microhemorrhage resulted in the suspension of the studies in 2012 [11]. Similarly, solanezumab (Eli Lilly), albeit resulted safe, showed a benefit that was not higher than that associated with the palliative acetylcholinesterase inhibitors drugs, and the studies were terminated [12]. Unfortunately, this has also recently been the case for aducanumab (Biogen Idec) [13]. However, other mAbs, such as gantenerumab (Hoffman-La Roche) and crenezumab (Genentech), are currently under phase III clinical trials [10].

Recombinant antibody fragments are reliable alternatives to the full-length antibodies from which they are derived [14,15]. These fragments retain the antigen-binding specificity of full-length mAbs and possess other interesting properties such as potential linkage to therapeutic payloads (enzymes or liposomes, etc.). The use of single chain antibody fragments (scFvs) is safer than that of full-length mAbs because scFvs lack the crystallizable fraction (Fc), which mediates microglia activation and subsequently induces pro-inflammatory cytokines and mediators secretion [11,16,17]. Similarly, the absence of the Fc fragment prevents scFvs from triggering of the complement system; therefore, they exhibit limited immunogenicity and prevent opsonization and antibody-dependent cell toxicity. Although scFvs have shorter half-lives than their corresponding full-length mAbs, they exhibit better tissue distribution, penetration, and clearance properties than the corresponding full-length mAbs [18].

In 2000, Frenkel *et al*. designed the first anti-Aβ scFv based on the variable regions of an anti-Aβ IgM [19]. Since then, some other recombinant engineered molecules have been developed [20] and modified using stabilizing mutations [21–23], including scFv-h3D6, an scFv derived from bapineuzumab [24]. ScFv-h3D6 has been incorporated with different mutations to enhance its thermodynamic stability for improving its pharmacokinetic parameters and thus its therapeutic effect [25]. A two-residue elongation of the C-terminal domain (V_L_) of scFv-h3D6 produced scFv-h3D6-EL, which shows 25% higher thermodynamic stability than the original scFv (referred to as scFv-h3D6-WT in this manuscript for clarity) [26]. *In vitro* studies showed that both scFv-h3D6 molecules prevented Aβ peptide-induced cytotoxicity, with scFv-h3D6-EL providing improved results [25]. Single administration of scFv-h3D6-WT in young 3xTg-AD female mice ameliorated the first hallmarks of AD by reducing amyloid burden and improving cognitive disabilities [27–29]. Next steps involve the elucidation of the mechanism underlying the protective effects of scFv-h3D6 to demonstrate the therapeutic potential of Aβ-immunotherapy and to lay the background for other studies on AD pathology in mouse.

In this study, we examined whether the higher thermodynamic stability of scFv-h3D6-EL than that of scFv-h3D6-WT improved its pharmacokinetic parameters. For this, 5-month-old 3xTg-AD female mice were administered scFv-h3D6-WT or scFv-h3D6-EL and plasma concentration profiles for these proteins were determined for elucidating their main pharmacokinetic parameters. Furthermore, some other factors such as the capability of scFv-h3D6 to cross the blood–brain barrier (BBB), its colocalization with the Aβ peptide, the specific cell types containing these molecules as well as its effect on tumor necrosis factor- α (TNF-α) levels were examined to determine the mechanism of action of scFv-h3D6.

## Materials and methods

### ScFv-h3D6 expression and purification

ScFv-h3D6 was recombinantly expressed in *Escherichia coli* and was purified, as described previously [24]. Lipopolysaccharide (LPS), a major endotoxin in gram-negative bacteria, was removed from the protein by using Detoxi-Gel Endotoxin Removing columns (ref. 20344, ThermoFisher Scientific, Waltham, MA, USA).

### Animals

A triple-transgenic mouse model of AD (3xTg-AD mouse model) was initially engineered at the University of California, Irvine. For this, two independent transgene constructs encoding human APP_Swe_ and tau_P301L_ proteins under the control of the mouse Thy1.2 promoter were co-microinjected into single-cell embryos harvested from mutant homozygous *PS1*_*M146V*_ knock-in mice [30,31]. *APP*_*Swe*_ and *PSEN1*_*M146V*_ mutations are associated with familial AD, whereas *MAPT*_*P301L*_ mutation is associated with a familial form of frontotemporal dementia and not with AD. This mouse model is shown to reproduce the human amyloid and tau pathologies observed in patients with AD through similar regional and temporal patterns [31,32]. The animals used in the present work belonged to the 3xTg-AD colony, and to the corresponding non-transgenic (NTg) mice (B6129SF2), established by our group at the UAB Animal Facility. Founder animals were provided by The Jackson Laboratory, Bar Harbor, ME, USA. Only female were used because they exhibit a greater Aβ burden and larger behavioral deficits than age-matched males [33]. Furthermore, in contrast to males, females maintain the original phenotypic traits described (https://www.jax.org/strain/004807).

The animals were maintained under standard laboratory conditions such as temperature of 22°C ± 2°C, relative humidity of 55% ± 5%, 12-/12-h light/dark cycle starting at 08:00 a.m., and bedding made of wood chips, and were given food and water *ad libitum*. All the experiments were approved by the UAB Animal Research Committee and the Government of Catalonia (Protocol Number: CEEAH-3553) and were conducted in accordance with the legislation for the protection of animals used for scientific purposes (directive 2010/63/EU; BOE 2013/1337). All efforts were made to minimize suffering.

### Experimental design

Two groups containing 24 randomly-distributed five-month-old 3xTg female mice each were intraperitoneally injected with 100 μg (~ 3.3 mg/kg) of scFv-h3D6-WT or scFv-h3D6-EL, diluted in 200 μl of vehicle (PBS-buffer, pH 7.4). These conditions were consistent with the already published experiments on scFv-h3D6-WT pharmacodynamics [27–29,34]. Animal sacrifices and sample collection were performed at the following time points p.i.: 0h (for PBS-administered mice), 1h, 5h, 10h, 1 day, 2 days, 5 days, and 8 days (n = 3).

### Sample collection

The animals were anesthetized by making them inhale 1% isoflurane. Firstly, blood was collected by cardiac puncture and was supplemented with a complete protease inhibitor cocktail (ref. 5892970001, Roche) and 1 mM EDTA to prevent protein degradation and coagulation. Next, the samples were centrifuged at 20,230 x *g* and 4°C for 10 min. The supernatant obtained was carefully recovered to prevent contamination from the interphase (white blood cells and platelets) and centrifuged again. Finally, the supernatant was recovered and stored at -80°C as blood plasma until further use. Next, the mice were guillotined and dissected, and their brains were quickly removed from their skulls, extensively rinsed in cold PBS, weighted, and dissected. Both hemispheres of the mouse brains were separately processed for histological and biochemical analyses.

### Protein extract preparation

The cerebral hemispheres were mechanically broken in a cold tissue homogenization buffer (TBS [pH 7.6] supplemented with the protease inhibitor cocktail and 1 mM EDTA) by using a tissue homogenizer (Sigma-Aldrich, Saint Louis, MO, USA). After gentle sonication with one cycle of 35 s, at 35% duty cycle, and output 4 by using Dynatech Sonic Dismembrator ARTEK 300; BioLogics INC., Manassas, VI, USA), the samples were centrifuged at 100,000 x *g* and 4°C for 1h. The supernatant obtained was stored at -80°C as the extracellular fraction. The pellet obtained was suspended in a lysis buffer (1% Triton X-100 in homogenization buffer), sonicated and centrifuged similarly, and the supernatant obtained was stored at -80°C as the intracellular fraction.

### Histological sections preparation

The tissues were immersed in 4% paraformaldehyde for 36h at 4°C. Next, the tissues were rinsed in PBS, dehydrated by immersing in a series of increasing concentrations of ethanol solutions (50%, 70%, 96%, and 100%), in xylene and 1:1 xylene:paraffin. Paraffin embedding was performed using the regular procedures from our laboratory. Coronal sections (10-μm thick) were mounted on Superfrost^TM^ Plus microscope slides (ref. 4951PLUS4, Thermo Fisher Scientific) and stored at R.T. until further use. The region of analysis corresponded to the range of coordinates between Fig 43 (interaural 2.34 mm and bregma -1.46 mm) and Fig 48 (interaural 1.74 mm and bregma -2.06 mm) in a study by Franklin and Paxinos [35].

### Anti-scFv-h3D6 production

#### Animal immunization

Anti-scFv-h3D6 polyclonal antibodies were produced by Cell Cultures, Antibody Production and Cytometry Core at the UAB. Two New Zealand rabbits were intradermally immunized with 400 μg of scFv-h3D6-WT protein conjugated with a Freund’s complete adjuvant. After the first immunization, the rabbits were administered three booster injections (at 21-day intervals) of 300 μg of scFv-h3D6-WT and Freund’s incomplete adjuvant. Blood samples were obtained from the rabbits to monitor serum-specific antibody levels by ELISA. Finally, total rabbit sera were collected 10 days after the fifth immunization.

#### Specificity analysis

Specificity of the anti-scFv-h3D6 polyclonal antibodies was determined by performing an ELISA standardized by the Cell Cultures, Antibody Production and Cytometry Core at the UAB. Briefly, all sera were analyzed using 96-well polystyrene plates (ref. 442404, Maxisorp, NUNC, Labclinics, Barcelona, Spain) coated with scFv-h3D6 diluted in 0.1 M carbonate buffer (pH 9.6). The ELISA plates were incubated with several dilutions of sera for 90 min and were washed extensively. Next, a peroxidase-conjugated anti-rabbit IgG to (ref. 170–6515, BioRad, Hercules, CA, USA) was added the plates to detect the antigen–antibody complexes. Immunodetection was performed using O-phenylenediamine dihydrochloride (OPD) Fast Substrate (ref. P9187; Sigma Aldrich), and absorbance was read at 450 nm by using a multilabel reader Victor3 (Perkin Elmer, Turku, Finland). S1 Fig shows the titration of the antibodies from the two animals. All subsequent experiments were performed using the hyperimmune serum obtained from rabbit 2 because it had a higher antibody titer than the serum obtained from rabbit 1.

### ScFv-h3D6 quantification

ScFv-h3D6 levels were quantified by indirect competitive ELISA. For this, 96-well polystyrene plates (ref. 44-2404-21, Maxisorp, NUNC, Labclinics) were coated with scFv-h3D6 (10 ng per well from a 100 ng/mL dilution in PBS, pH 7.4) and were incubated overnight at 4°C. After several washes with 0.1% Tween-20 in PBS, the plates were blocked with 1% bovine serum albumin (BSA) in PBS for 30 min at 37°C and were washed again. Simultaneously, the samples (6 replicas for each mouse (n = 3) and time point) were incubated with the primary anti-scFv-h3D6 antibody (dilution 1:100,000) at 37°C for 1h and were added to the already blocked plates for 1h at 37°C. Next, the plates were washed again. The primary antibody was detected by incubating the plates with a peroxidase-conjugated anti-rabbit IgG secondary antibody (ref. 1662408, dilution 1:2000, BioRad) for 1h at 37°C and was revealed and visualized using the OPD Fast Substrate. Absorbance was read at 450 nm by using the multilabel reader Victor3. Values were obtained using specific calibration curves for scFv-h3D6-WT and scFv-h3D6-EL each. The titration took into account and annulated putative differences in the binding of the anti-scFv-h3D6 antibodies to the two molecules.

### Aβ peptide quantification

Aβ_42_ peptide levels were quantified from 6 replicas for each mouse (n = 3) and time point and using a commercial amyloid-β 42 ELISA kit (ref. KMB3441, Invitrogen), according to the manufacturer’s protocol. Absorbance was measured at 450 nm by using the multilabel reader Victor3. Data obtained were normalized using the total amount of protein in each extract, as measured using the bicinchoninic acid (BCA) assay (ref. 23225, Pierce, ThermoFisher Scientific).

### TNF-α quantification

TNF-α levels in hippocampal protein extracts were quantified from 6 replicas for each mouse (n = 3) and time point and using a commercial ELISA DuoSet kit (ref. DY410-05, R&D Systems, Minneapolis, MN, USA). Absorbance was measured using the multilabel reader Victor3. Because wavelength correction was not available, readings obtained at 540 nm were subtracted from those obtained at 450 nm. Next, the data obtained were normalized using the total amount of protein in each extract, as measured by the BCA assay.

### Fluorescent immunodetection and colocalization

The tissue sections were deparaffined in xylene, hydrated in decreasing concentrations of ethanol solutions, and extensively washed in distilled water. Non-binding sites were blocked using 5% BSA in PBS containing 0.05% Tween-20 and 5% normal goat serum for 1h at room temperature. Next, the sections were incubated overnight at 4°C with the anti-scFv-h3D6 polyclonal antibody (dilution 1:100, produced by the Cell Cultures, Antibody Production and Cytometry Core, UAB), washed several times, and incubated with Alexa 488 fluorophore-conjugated goat polyclonal anti-rabbit secondary antibody (ref. ab150077, dilution, 1:200 Abcam, Cambridge, UK) for 1h at R.T. Finally, the sections were cover-slipped for microscopic observation with 4’, 6-diamino-2-phenylindole (DAPI)-containing Vectashield solution (ref. D9542, Vector Laboratories, Burlingame, CA, USA) for fluorescent nuclei staining. Immunostaining controls were generated using a blocking buffer instead of the primary antibodies. The specificity of the anti-scFv-h3D6 antibody was determined based on the absence of immunofluorescent staining in non-injected controls (vehicle-injected mice corresponding to 0h p.i.).

Double immunodetection of scFv-h3D6 and Aβ peptide was performed by incubating the samples with anti-scFv-h3D6 antibody and 6E10 mAb (ref. SIG-39320, dilition1:100, Covance Signet, Princeton, NJ, USA), followed by incubation with Alexa 488-conjugated anti-rabbit and Cy3-conjugated goat polyclonal anti-mouse secondary antibodies (ref. AP124C, dilution 1:100, Chemicon, Millipore, Billerica, MA, USA), respectively.

### Epifluorescence and confocal microscopy

Photographs were digitally captured using ProgRes C10^*Plus*^ color video camera coupled with a Zeiss Axiosphot microscope by using ProgRes Capture Pro program (Jenoptik, Jena, Germany). Confocal microscopy was performed at the Microscopy Facility of the UAB using a Spectral Fluoview-1000 device (Olympus, Shinjuku, Tokio, Japan) and capturing 5–10 optical sections at 1-μm intervals with an objective lens UPLSAO0. The obtained images were processed and assembled with Image J software (v. 1.43 u, NIH).

## Results

### Comparison of the pharmacokinetic parameters of scFv-h3D6-WT and scFv-h3D6-EL

The beneficial effects of a single intraperitoneal injection of 100 μg scFv-h3D6 have already been demonstrated in young 3xTg-AD female mice [27–29]. In this work, we analyzed the pharmacokinetic parameters of scFv-h3D6 by studying variations in the plasma concentrations of the original version of the molecule (scFv-h3D6-WT) and its elongated and thermodynamically more stable version (scFv-h3D6-EL) [24,26].

**Fig 1:**
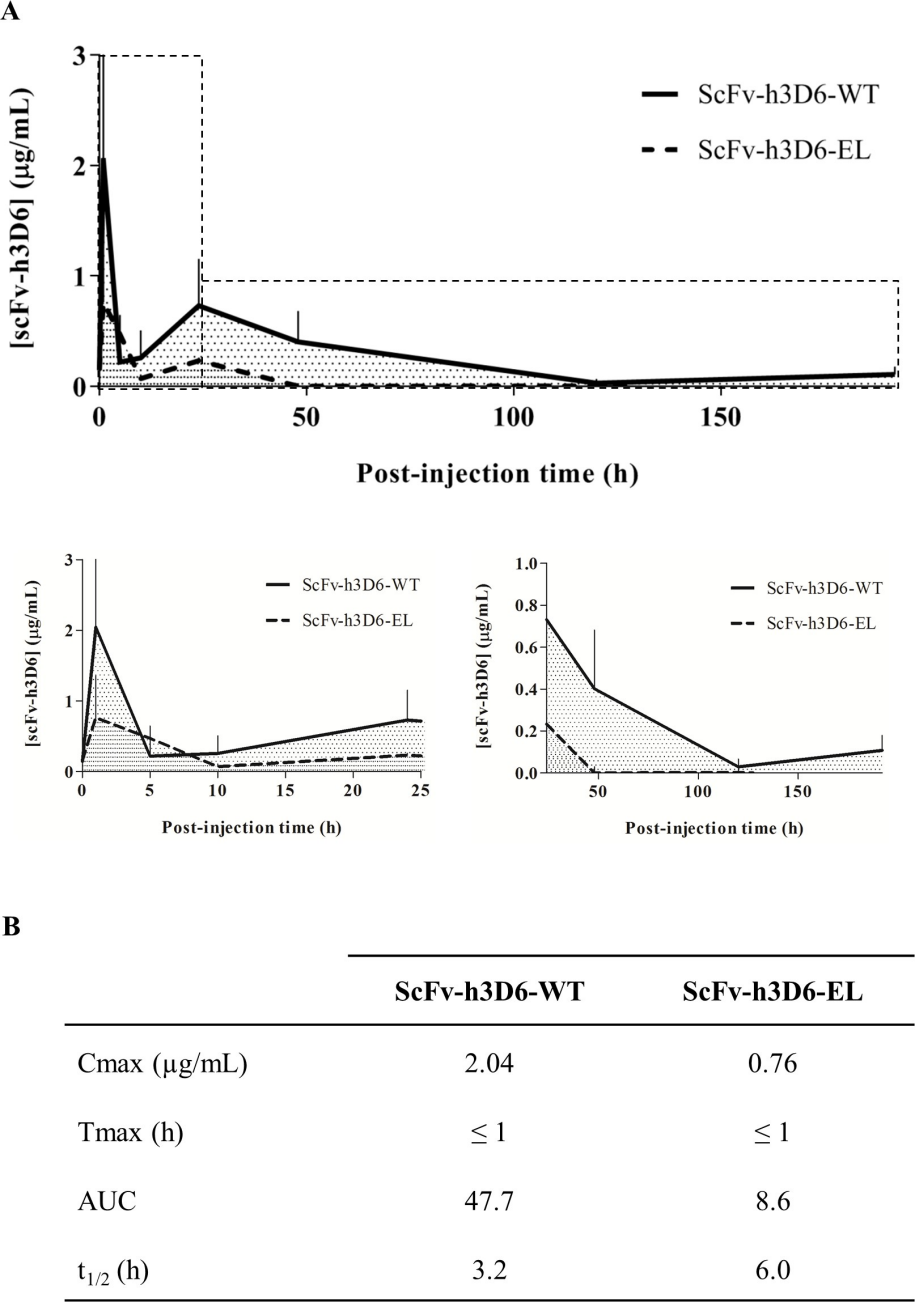
Plasma concentrations of scFv-h3D6-WT and scFv-h3D6-EL. (A) Plasma concentrations of scFv-h3D6-WT and scFv-h3D6-EL were measured by competitive ELISA at 0h (for vehicle-injected mice), 1h, 5h, 10h, 1 day, 2 days, 5 days, and 8 days p.i. The continuous line indicates the scFv-h3D6-WT profile, and the dashed line the scFv-h3D6-EL profile. Insets below the plot are magnifications for early (0-25h) and late (25-192h) p.i. times. The concentrations are expressed as micrograms of scFv-h3D6 per milliliter of the plasma and are represented as means and SEM. (B) Comparative pharmacokinetic parameters of scFv-h3D6-WT and scFv-h3D6-EL were quantified using the concentration–time curve; Cmax (maximum plasma concentration, μg/mL), Tmax (time to reach Cmax, h), AUC (area under the curve), and t_1/2_ (half-life, h).

The plasma concentrations of these molecules were quantified at 0h, 1h, 5h, 10h, 1 day, 2 days, 5 days, and 8 days post-injection (p.i.) by performing competitive enzyme-linked immunosorbent assay (ELISA) by using specific calibration curves for each molecule. Values are presented by plotting a concentration–time curve (Fig 1A), from which the main pharmacokinetic parameters of these molecules were calculated (Fig 1B).

Both molecules were absorbed from the intraperitoneal cavity into the blood before 1h p.i. (Fig 1A) and showed a similar profile. However, a higher peak was initially observed for scFv-h3D6-WT than for scFv-h3D6-EL, indicating enhanced absorption of the original molecule. Following the pronounced initial peak, a second peak appeared at ~24h p.i., suggesting a two-phased absorption mode or a compartmental distribution of both molecules. The higher absorption of scFv-h3D6-WT than that of scFv-h3D6-EL affected the remaining pharmacokinetic profile, with area under the curve (AUC), which were measured using a trapezoidal rule for n-1 subintervals from 0 to the last time point, of scFv-h3D6-WT being five-fold higher than those of scFv-h3D6-EL (Fig 1B). However, scFv-h3D6-EL exhibited a longer half-life than the scFv-h3D6-WT, with the values for scFv-h3D6-EL being almost two-times higher than those for scFv-h3D6-WT, which was consistent with the improved stability of scFv-h3D6-EL observed in biophysical analyses and in cell cultures [25,26]. Because of the difference in the absorption of scFv-h3D6-WT and scFv-h3D6-EL, subsequent experiments were performed using the original version of the molecule.

### ScFv-h3D6 can cross the blood–brain barrier

**Fig 2:**
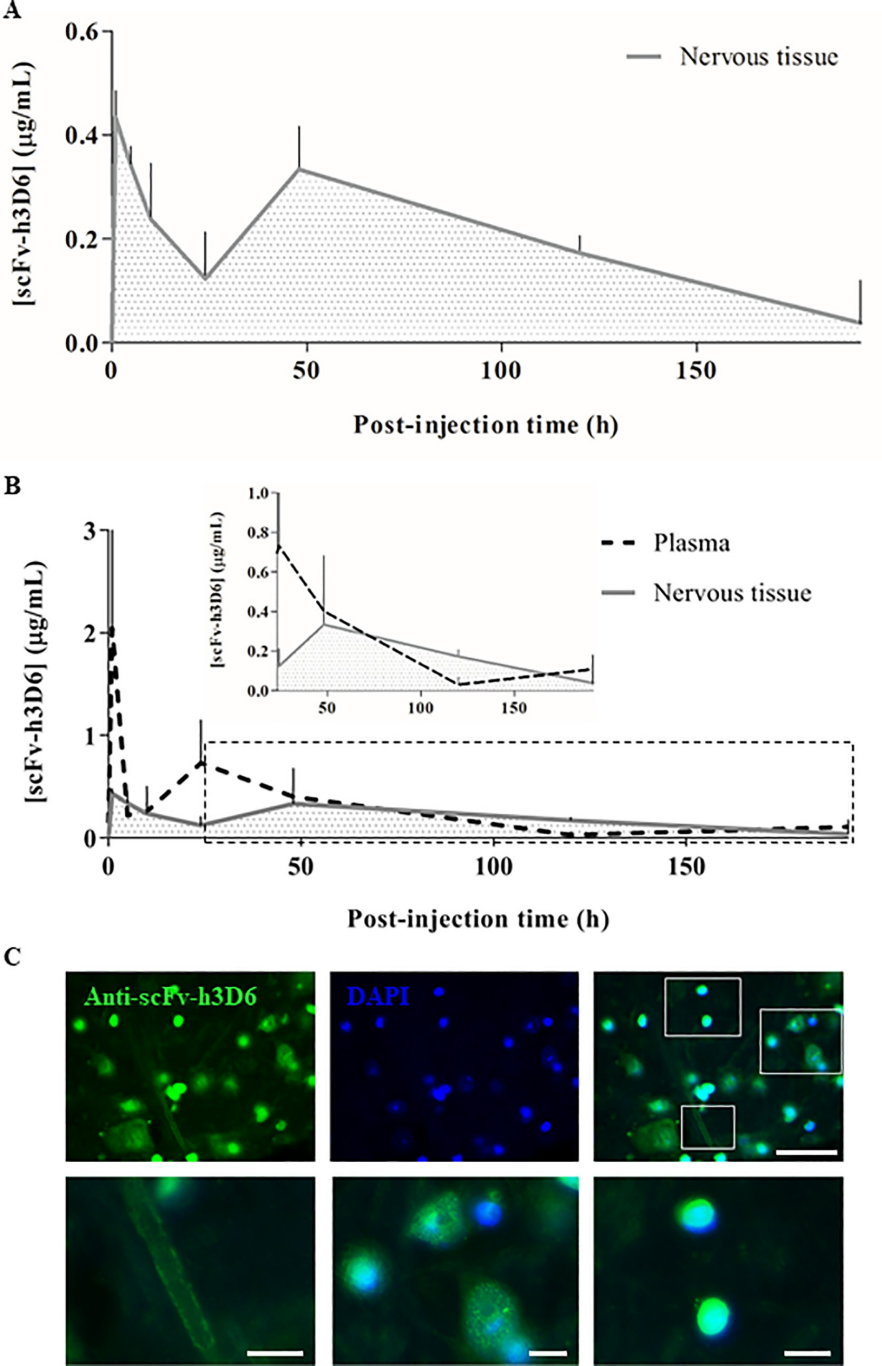
ScFv-h3D6 influx into the brain. ScFv-h3D6 was detected and quantified by ELISA. (A) ScFv-h3D6 concentration in the nervous tissue protein extracts over time p.i.: 0h (for vehicle-injected mice), 1h, 5h, 10h, 1 day, 2 days, 5 days, and 8 days. (B) Comparative profiles of scFv-h3D6 in the plasma (dashed line) and nervous tissue (grey area). The inset within the plot magnifies late p.i. times (25-192h). The concentrations are expressed as microgram of scFv-h3D6 per milliliter of the plasma or brain protein extract and are represented as means and SEM. (C) Representative illustration, corresponding to the amygdalar region showing scFv-h3D6 in a 10-μm-thick brain section (at 5h p.i.). On the top from the left to the right: scFv-h3D6 (in green), DAPI (in blue), and merged image. Scale bar, 50 μm. Squares in the merged image are shown at the bottom and correspond to a magnified blood vessel, large neurons, and small cells. Scale bars, 10 μm.

To further determine whether scFv-h3D6 could penetrate the central nervous system, we determined the concentration of this molecule in the brain at different time points p.i. by competitive ELISA with protein extracts and by fluorescent immunodetection with histological sections. ScFv-h3D6 concentration in the brain protein extracts over time p.i. produced a main peak at the very beginning (~1h p.i.) and a slightly smaller peak later (~48h p.i.), after which it decreased progressively until the end of the experiment (8 days p.i.; Fig 2A). These results were compared with the plasma concentration of scFv-h3D6 to determine the temporality of its distribution through the central nervous system (Fig 2B). Consistently, the first peak of scFv-h3D6 in the plasma overlapped the first peak in the brain, suggesting that the fast absorption of the molecule from the intraperitoneal cavity into the blood coincided with its early distribution to other tissues such as the central nervous system. The increased plasma concentration of scFv-h3D6 at ~24h, which produced a second peak in its plasma profile, produced a second peak in its central nervous system profile at 48h p.i. These observations can be explained by the progressive re-distribution of plasma scFv-h3D6 (probably more slowly than its initial distribution because of its decreased concentration).

Fig 2C illustrates the immunodetection of scFv-h3D6 in the amygdalar region of a mouse sacrificed at 5h p.i. as a representative of its early entry into the central nervous system. A fluorescent signal was observed throughout the tissue, with increased signal intensities observed mainly around the blood vessel walls and in the cell body of both large and small cells (Fig 2C, bottom).

The first location suggests the accumulation of the molecule through the BBB as a momentary influx into/efflux from the central nervous system or because of its deposition tendency along the BBB. Detection of the fluorescent signal in large cells indicated that scFv-h3D6 was internalized by large neurons, and detection of a strong signal intensity in the small cells indicated that scFv-h3D6 was internalized by glial cells or small neurons (see Section 2.5). The fact that the signal was observed within the cell body indicated that the penetration of scFv-h3D6 into the brain was actual and was not associated with the presence of a small amount of blood remaining in the vessels.

### ScFv-h3D6 is internalized by cells

**Fig 3:**
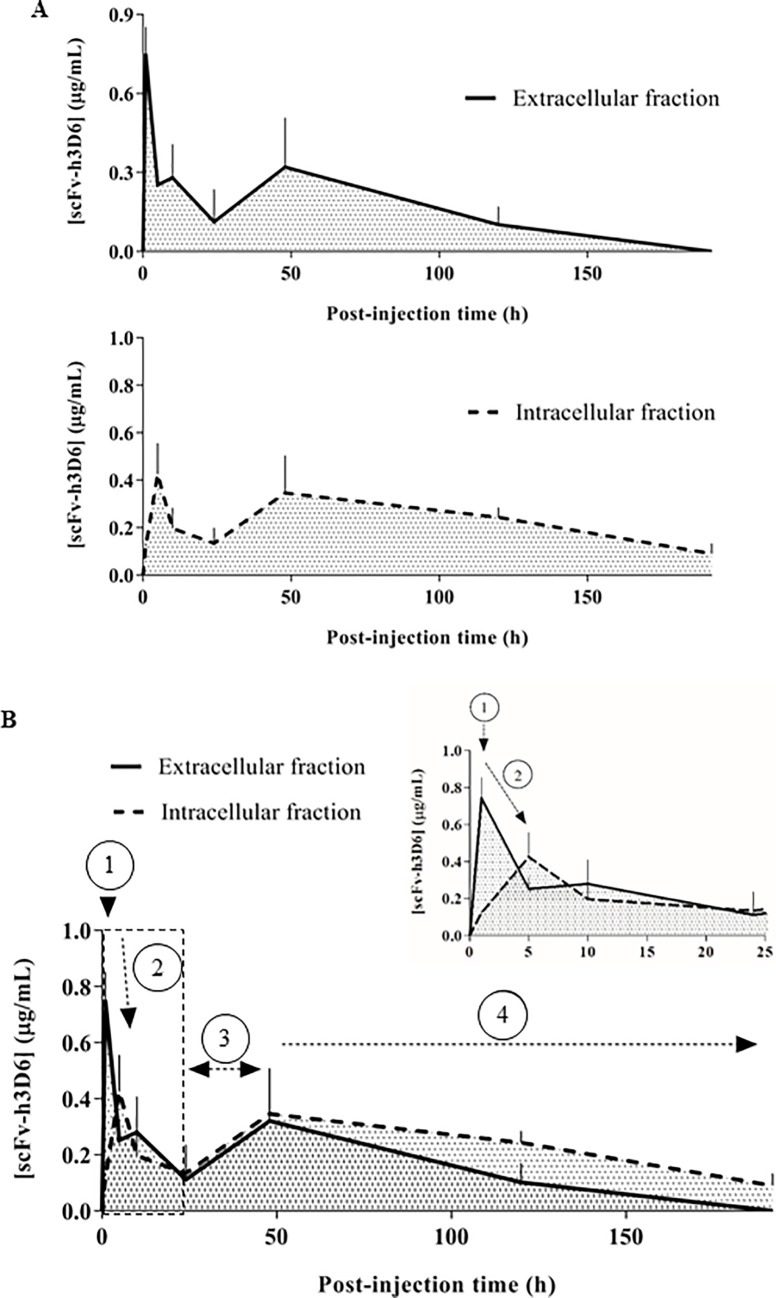
Temporal distribution of scFv-h3D6 through the extra- and intracellular brain compartments. (A) Extracellular (continuous line, top) and intracellular (dashed line, bottom) scFv-h3D6 levels are represented over time p.i.: 0h (for vehicle-injected mice), 1h, 5h, 10h, 1 day, 2 days, 5 days, and 8 days. The concentrations are expressed as microgram of scFv-h3D6 per milliliter of the brain protein extract. (B) Comparative superposition of both extra- and intracellular profiles of scFv-h3D6. The first peak in scFv-h3D6 concentration in the extracellular compartment appeared 1h p.i. and indicated the penetration of scFv-h3D6 into the nervous tissue (1). The first peak in scFv-h3D6 concentration in the intracellular compartment appeared later at 5h p.i. and coincided with the reduction in the extracellular levels of scFv-h3D6, suggesting the cellular internalization of the molecule (2). In both compartments, a second peak in scFv-h3D6 concentration appeared later at 48h p.i. probably due to the influx of the scFv-h3D6 present in the plasma at 24h p.i. (3). The clearance process was evidenced by the progressive decrease in scFv-h3D6 concentration from 48h to the end of the experiment (4). The inset corresponds to the amplification of the initial profile up to 25h p.i.

Next, we quantified scFv-h3D6 levels in the extra- and intracellular brain compartments to further determine the process underlying its internalization. Protein extracts of samples obtained at the same time points p.i. were analyzed by competitive ELISA. Fig 3A shows scFv-h3D6 concentration in the extra- and intracellular compartments over time. The first peak in scFv-h3D6 concentration in the extracellular compartment was almost two-times higher than its first peak in the intracellular compartment; however, differences in scFv-h3D6 concentration between these compartments were not as significant at the remaining time points p.i. This suggested that the early inflow of scFv-h3D6 into the central nervous system promoted its initial accumulation in the extracellular compartment and subsequent distribution in both extra- and intracellular compartments.

Comparative superposition of both the extra- and intracellular profiles of scFv-h3D6 facilitated the interpretation of our results (Fig 3B). The first peak in scFv-h3D6 concentration in the extracellular compartment (~1h p.i.) indicated its penetration into the central nervous system. The first peak in scFv-h3D6 concentration in the intracellular compartment appeared later (~5h p.i.) and coincided with its reduction in the extracellular levels, suggesting its internalization. A second and faint peak in scFv-h3D6 concentration appeared at ~48h p.i. in both compartments, which may have been caused by the influx of scFv-h3D6 from the plasma at 24h p.i., as suggested in the previous section (Section 2.2, Fig 2B). Thereafter, scFv-h3D6 concentration progressively decreased in both compartments, indicating its clearance. Thus, scFv-h3D6 concentration in the intracellular compartment was higher than that in the extracellular compartment throughout the late phase p.i., suggesting a cell-mediated mechanism underlying scFv-h3D6 clearance in the brain.

### Compartmentalization of scFv-h3D6 and Aβ peptide in the central nervous system

**Fig 4:**
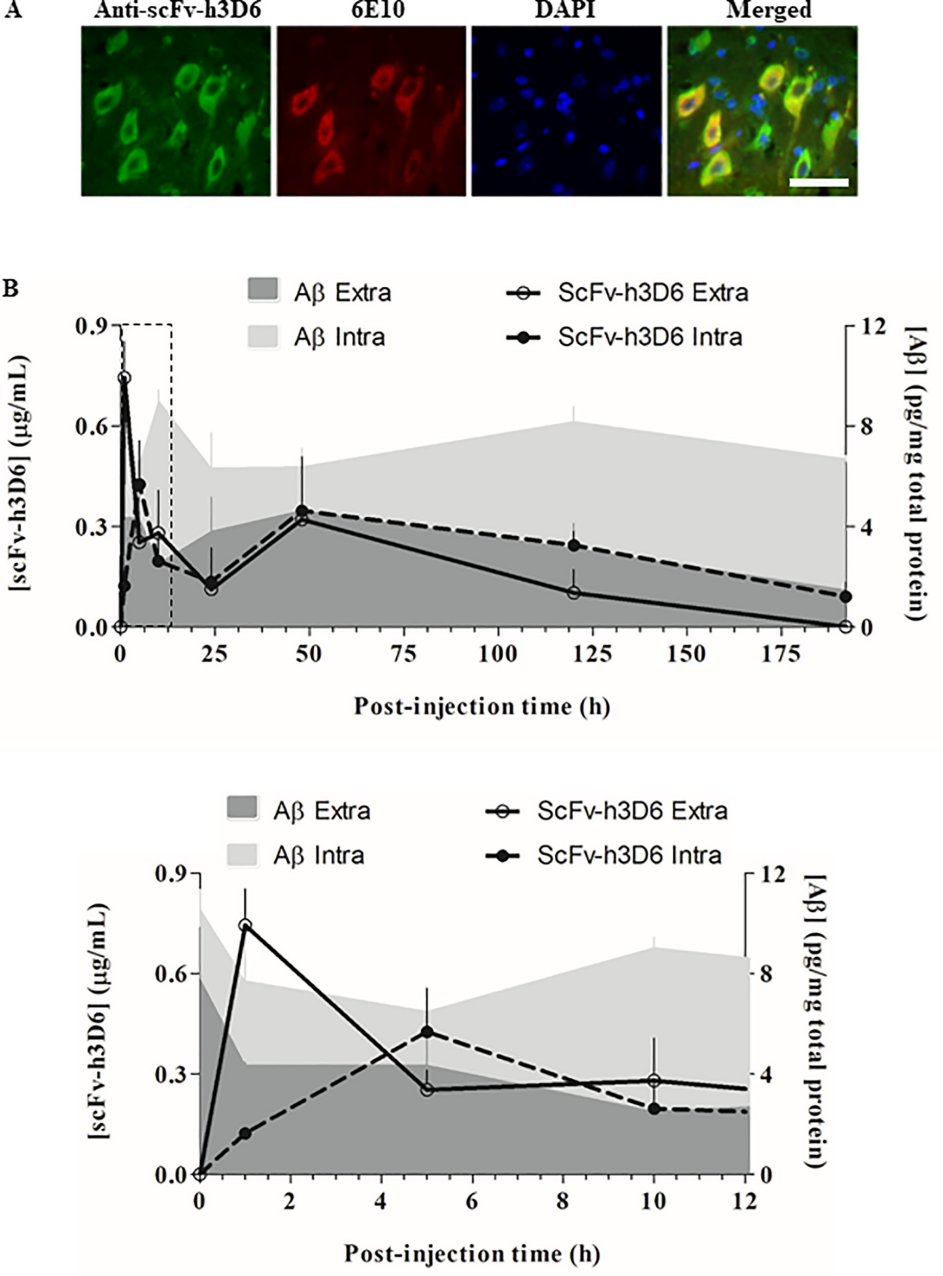
Colocalization of scFv-h3D6 and Aβ peptide in the brain. (A) Colocalization of scFv-h3D6 and Aβ peptide in a 10-μm-thick brain section (at 5h p.i.). Scale bar, 100 μm. (B) Extracellular and intracellular levels of Aβ peptide and scFv-h3D6 over time p.i.: 0h (for vehicle-injected mice), 1h, 5h, 10h, 1 day, 2 days, 5 days, and 8 days. The inset corresponds to the amplification of the initial profiles up to 12h p.i. ScFv-h3D6 concentration is expressed as microgram of scFv-h3D6 per milliliter of the brain protein extract. Aβ peptide concentration is expressed as picogram of Aβ peptide per milligram of total protein in the extract.

Because the therapeutic effect of scFv-h3D6 is mediated by a reduction in Aβ peptide burden [28,36], we examined the interaction between these both molecules in terms of compartmental distribution (extra- and intracellular compartments) and temporary evolution (by comparing their concentrations over time). First, scFv-h3D6 and Aβ peptide were immunodetected in the histological sections of a mouse sacrificed at 5h p.i., which coincided with the main peak in scFv-h3D6 concentration in the intracellular compartment (Fig 4A). A strong signal for both molecules was detected in the intracellular compartment; however, not all cells showing the scFv-h3D6 signal contained the Aβ peptide. Moreover, scFv-h3D6 was immunodetected within large and small cells, whereas the Aβ peptide was mostly detected in only large cells.

Aβ burden was also quantified using a commercial ELISA kit and was compared with the scFv-h3D6 concentration profiles in both extra- and the intracellular compartments. Fig 4B and 4C shows Aβ peptide concentration over time p.i., and its comparison with the scFv-h3D6 concentration. The first response to the treatment is shown in detail in panels showing the magnification of the 0–12h p.i. interval (see insets). Aβ peptide concentration was higher in the intracellular compartment than in the extracellular compartment at all the analyzed time points p.i., which was consistent with the early stage of the disease in the 5-mo-old 3xTg-AD mouse model [37,38]. The extracellular Aβ peptide concentration exhibited a pronounced decline tendency during the early phase p.i. (0–10h) and a subsequent progressive decrease later (from 2 days p.i.), which overlapped with the main first peak and the faint second peak in scFv-h3D6 concentration, respectively (Fig 4C). Aβ peptide concentration in the intracellular compartment reduced pronouncedly after the first peak in scFv-h3D6 concentration (0–5h p.i.). Interestingly, Aβ peptide concentration in the intracellular compartment increased at 10h and 5 days p.i., which coincided with the decrease in Aβ peptide concentration in the extracellular compartment, suggesting the engulfment of extracellular Aβ peptide.

### Colocalization of scFv-h3D6 and Aβ peptide in different cell types

**Fig 5:**
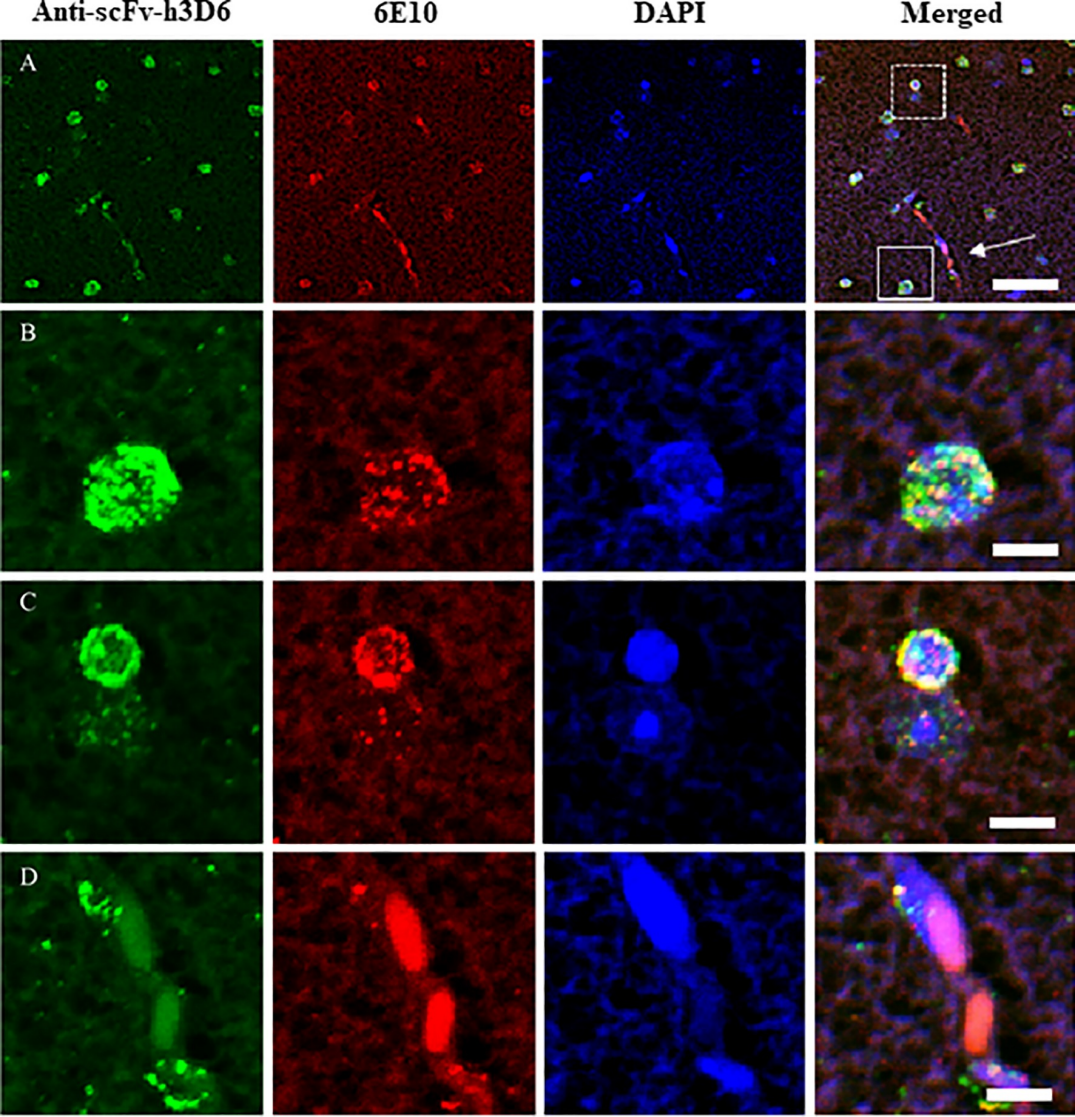
ScFv-h3D6 and Aβ peptide visualization 5 days p.i. by confocal microscopy. Colocalization of scFv-h3D6 and Aβ peptide in a 10-μm-thick brain section (at 5 days p.i.). (A) Panoramic image. Continued-line square in the merged figure shows an example of a large cell containing both scFv-h3D6 and Aβ peptide signals, which have been magnified in (B). Dashed-line square shows an example of a couple closely related cells, which have been magnified in (C). White arrow indicates the area next to the blood vessel, which have been magnified in (D). Scale bars: 50 μm in (A) and 10 μm in (B), (C), and (D).

Although scFv-h3D6 and Aβ peptide partially colocalized at the early phase after the treatment (at 5h p.i., as shown in the previous section; Fig 4A), we found that scFv-h3D6 was localized within both large and small cells whereas the Aβ peptide signal was only localized within the large cells. Fig 5A shows a representative panoramic image obtained by confocal microscopy of a mouse sacrificed 5 days p.i. to determine scFv-h3D6 and Aβ peptide colocalization and cell types involved at advanced time points p.i. Fig 5B–5D shows the cellular casuistry observed in the panoramic image, namely, (B) solitary cells, (C) couple of adjacent cells, and (D) cells located around blood vessels. Fig 5B shows a large solitary cell containing both scFv-h3D6 and Aβ peptide. Fig 5C shows a couple of adjacent cells conformed by a large cell with a very low signal for both scFv-h3D6 and Aβ peptide and a small cell with strong signal for both scFv-h3D6 and Aβ peptide. Fig 5D shows a couple of cells located around the blood vessels with a higher signal for scFv-h3D6 than for the Aβ peptide.

Cell types were determined using a recently reported algorithm, based on the cytological features [39]. Thus, large cells were defined as large neurons because of their large-sized and lightly stained nucleus, with an “empty” appearance, and small cells were defined as glial cells because their small and darkly stained nucleus. Similarly, endothelial cells were clearly distinguished based on the shape of their nuclei wrapping around blood vessels. Although specific immunostaining is the most suitable methodology to classify cell types, we are confident that glial cells are actual because we previously showed them by glial fibrillary acidic protein (GFAP) and ionized calcium binding adaptor molecule 1 (Iba1) immunostaining [38].

Thus, the overall data suggested that in the early phase p.i. scFv-h3D6 was internalized by large neurons containing the Aβ peptide and was engulfed by glial cells as a part of the clearance process. Interestingly, in the late phase p.i., both scFv-h3D6 and Aβ peptide were strongly immunodetected within solitary large neurons but were almost negligible when these large neurons were accompanied by adjacent glial cells; however, a strong signal for scFv-h3D6 and Aβ peptide was detected in these glial cells. This suggested that phagocytic glial cells engulfed the intraneuronal Aβ peptide/scFv-h3D6 complex extruded into the extraneuronal compartment by large neurons. Fig 5D shows a couple of endothelial cells located around blood vessels containing both scFv-h3D6 and Aβ peptide. This indicates the involvement of endothelial cells in the flux of scFv-h3D6 and the Aβ peptide/scFv-h3D6 complex across the BBB.

### Effect of scFv-h3D6 treatment on TNF-α levels

**Fig 6:**
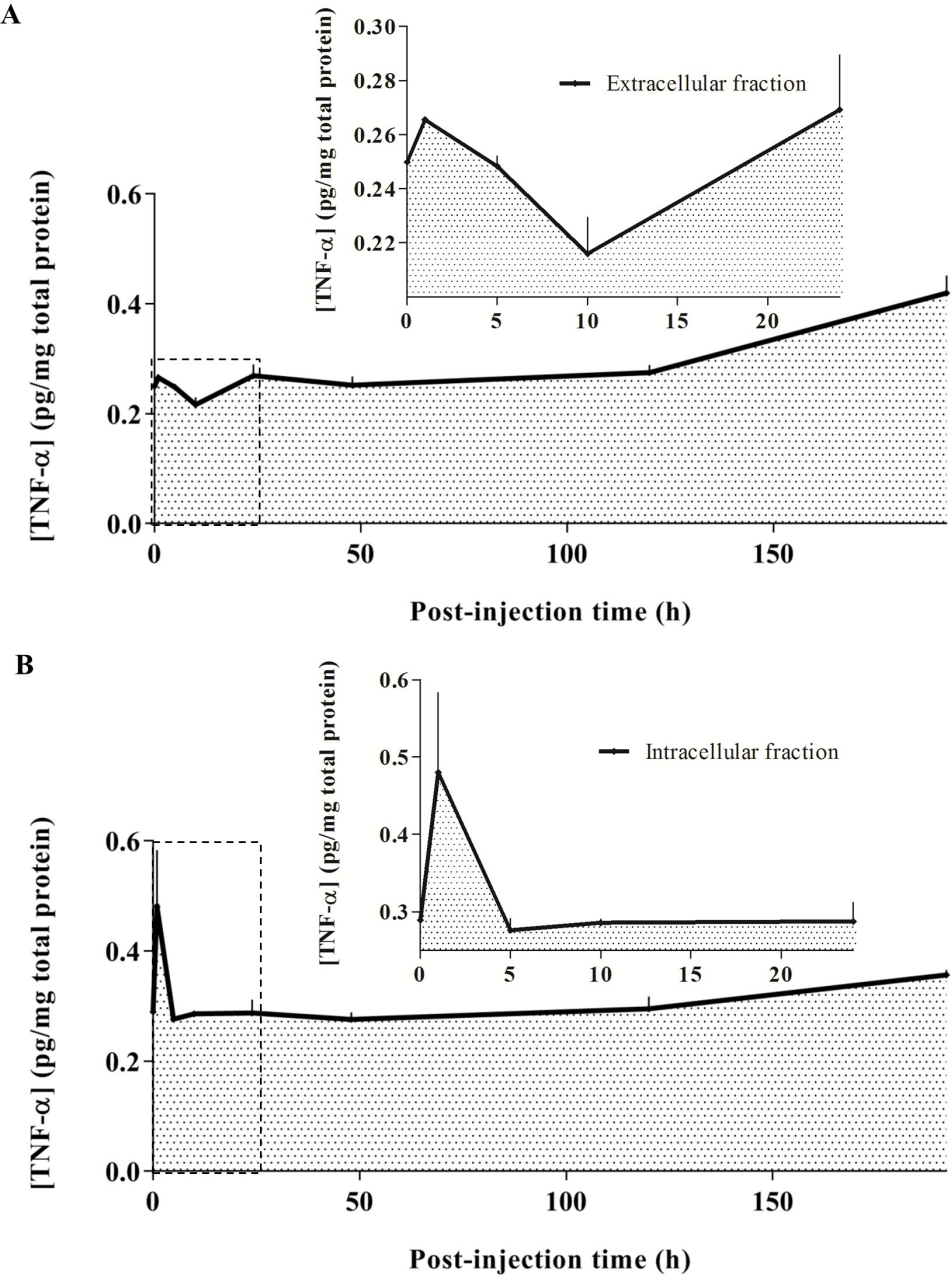
TNF-α profile over time post-injection. (A) Extracellular and (B) intracellular concentrations (pg/mg of total protein) of TNF-α are represented over time p.i.: 0h (for vehicle-injected mice), 1h, 5h, 10h, 1 day, 2 days, 5 days, and 8 days. The insets correspond to the amplification of the initial profile up to 24h p.i.

Because glial cells are involved in the clearance of scFv-h3D6 and the Aβ peptide/scFv-h3D6 complex and it is known that direct stimulation of glial cells may produce an early and critical response in terms of TNF-α levels increase [40], we measured levels of this cytokine in both extra- and intracellular compartments (Fig 6). We found that TNF-α levels increased in the intracellular compartment as a very early punctual response (only at ~1h p.i.). However, TNF-α levels increased during the late phase in both compartments, which was consistent with the occurrence of the clearance process in this phase.

## Discussion

At present, only palliative treatment is available for AD, which interferes with the symptoms rather than the causes of AD and therefore cannot preclude the dramatic deterioration of patients with AD [2,8,9]. Determination of disease-modifying treatments involves enormous efforts and considerations for determining appropriate targets and mechanisms of action [41]. Molecular engineering and treatment designing play a key role in determining the suitability of a therapy. ScFv-h3D6 is already known to improve the first hallmarks of AD [27–29]. Moreover, molecular redesigning produced an elongated and thermodynamically more stable version of scFv-h3D6, i.e., scFv-h3D6-EL [25,26]. In the present study, we determined the pharmacokinetic parameters of scFv-h3D6 (scFv-h3D6-WT) and compared them with those of the thermodynamically improved version (scFv-h3D6-EL) to test whether the *in vitro* benefits of greater stability [25,26] were maintained *in vivo*. ScFv-h3D6-WT showed better absorption from the intraperitoneal cavity into the blood than scFv-h3D6-EL, whereas scFv-h3D6-EL exhibited a longer half-life than scFv-h3D6-WT. This indicated that the improved stability of scFv-h3D6-EL translated into its *in vivo* performance but its modified structure hindered its absorption, thus reducing its potential effects. Moreover, these results also highlighted the reciprocal requirement between pharmacokinetic parameters and molecular/treatment designing because, for example, a different administration route could be used to improve scFv-h3D6-EL absorption [42].

Determination of the mechanism of action of a drug is also important for its molecular designing to improve the efficiency and safety of the drug. ScFv-h3D6 prevents neuron loss and improves cognitive impairment in young 3xTg-AD female mice by reducing intracellular Aβ peptide levels [27–29]. However, the mechanism underlying the reduction in Aβ peptide levels is unclear. At least three mechanisms have been proposed to explain the reduction in amyloid burden in the brain by anti-Aβ antibodies. The first mechanism suggests that the antibodies enter the brain and disaggregate amyloid fibrils by binding to the peptide [43–45], i.e., the N-terminal region of Aβ peptide in the case of scFv-h3D6. The second mechanism suggests that the antibodies cross the BBB and opsonize amyloid fibrils to activate the immune system, thus enhancing clearance (via microglia activation and Fc-mediated phagocytosis) [46–48]. The third mechanism (known as the sink hypothesis) is based on the peripheral Aβ peptide sequestering by the administered antibodies that modifies the brain–periphery equilibrium, thus promoting Aβ peptide efflux from the brain into the plasma [49]. All the three mechanisms are supported by *in vivo* data. Moreover, it has been described that epitope specificity of the Aβ-antibodies determines their mode of action [50]. An additional hypothesis, the masking hypothesis, by which binding of anti-Aβ scFvs to Aβ would be sufficient to reduce neurotoxicity [51], cannot be discarded. However, this does not seem to be the case for scFv-h3D6 since it was intracellularly colocalized with the Aβ peptide in this work.

In PDAPP mice, peripherally injected m3D6 mAb enters the brain and binds to amyloid plaques to induce Fc-gamma receptor-mediated microglia phagocytosis of the Aβ deposits [48]. The humanized version of the m3D6 mAb, i.e., bapineuzumab, also crosses the BBB and binds to a variety of Aβ species in the brain. However, other mAbs (such as solanezumab and crenezumab) inefficiently bind to the Aβ peptide in the human brain despite their efficacy in mice probably because of the lack of specificity due to cross-reactivity with other proteins showing epitope overlap [52]. In this work, we observed that scFv-h3D6 crosses the BBB as expected because the low molecular weight of scFvs is suggested to facilitate their passage across the BBB [23] and because other scFvs have already been detected in the brain [53].

Once in the brain, scFv-h3D6 was internalized by large neurons or engulfed by glial cells because it was immunodetected in both cell types. It is more likely that the neurons internalizing scFv-h3D6 are those containing the Aβ peptide because both molecules were colocalized within large neurons. Interestingly, the intracellular scFv-h3D6 signal showed a punctuate pattern similar to that observed for the Aβ peptide. Because we previously showed that Aβ colocalized with cathepsin D, a marker for intracellular vesicular structures [38], and here we show colocalization of Aβ and scFv-h3D6, it is tentative to suggest the vesicular-mediated internalization of scFv-h3D6. However, future studies are needed to determine the mechanism underlying the internalization of this molecule, which could be related to the already demonstrated neuronal internalization of the Aβ peptide involving lipid rafts and lipid raft-associated receptors or other modes [54].

Glial cells mainly contained scFv-h3D6 but not the Aβ peptide in the early phase p.i. and both scFv-h3D6 and Aβ peptide in the late phase p.i., thus highlighting potential mechanisms for a couple of processes, including scFv-h3D6 clearance in the brain and scFv-h3D6-mediated Aβ peptide withdrawal. On the one side, glial cells containing only scFv-h3D6, which were mainly observed in the early phase p.i. (~5h p.i.), may explain glial engulfment of scFv-h3D6 as the pharmacokinetic mechanism underlying its clearance. On the other side, glial cells containing both scFv-h3D6 and Aβ peptide, which were observed in the late phase p.i. (~5 days p.i.), may explain glial engulfment of the extruded Aβ peptide/scFv-h3D6 complex as the mechanism underlying Aβ peptide withdrawal. The Aβ peptide/scFv-h3D6 complex extruded from the intraneuronal compartment and its subsequent engulfment by glial cells could contribute importantly to neuronal homeostasis and survival. In addition, brain TNF-α levels showed an acute peak in the very early phase p.i. (~1h p.i.) and a progressive increase in the late phase p.i. (from ~5 days p.i. up to the end of the experiment), suggesting an initial response corresponding to scFv-h3D6 engulfment and later phagocytosis of the Aβ peptide/scFv-h3D6 complex [55].

In conclusion, determination of the pharmacokinetic profile of scFv-h3D6 showed that the thermodynamically stable scFv-h3D6-EL had a longer half-life than the original scFv-h3D6-WT as expected but showed strikingly impaired absorption. Moreover, the pharmacokinetic profiles of both scFv-h3D6-EL and scFv-h3D6-WT and the subsequent brain analyses of scFv-h3D6-WT showed that scFv-h3D6 crossed the BBB and entered neurons to capture the Aβ peptide, which was likely cleared from the brain through the engulfment of the Aβ peptide/scFv-h3D6 complex by glial cells.

## Supporting information

S1 Fig Titration of the anti-scFv-h3D6 antibodies. (A) Sera from the two immunized rabbits were analyzed. Pre-serum (dashed lines) and hyperimmune serum (continuous lines) from rabbit 1 (on the left) and rabbit 2 (on the right). (B) A standard curve fitting to a 4PL curve. Antibody titer (1/IC_50_) in the hyperimmune serum from rabbit 2 (dashed line) was higher than that in the serum from rabbit 1 (continuous line) and therefore was selected for performing subsequent experiments.
(TIF)

## Abbreviations

Aβ: amyloid-beta
AD: Alzheimer’s disease
APP: amyloid precursor protein
AUC: area under the curve
BBB: blood–brain barrier
BCA: bicinchoninic acid
BSA: bovine serum albumin
DAPI: 4’,6-diamino-2-phenilindol
EDTA: ethylenediaminetetraacetic acid
ELISA: Enzyme-Linked ImmunoSorbent Assay
Fc: crystallizable fraction
FcγR: Fc gamma receptor
mAb: monoclonal antibody
MAPT: microtubule-associated protein tau
mo: months-old
NTg: non-transgenic
OPD: o-Phenylenediamine dihydrochloride
PBS: phosphate-buffered saline
PS1/*PSEN1*: presenilin 1 protein/gene
p.i.: post-injection
scFv: single-chain variable fragment
scFv-h3D6-EL: elongated version of the humanized 3D6-derived scFv
scFv-h3D6-WT: original version of the humanized 3D6-derived scFv
TBS: Tris-buffered saline
TNF-α: tumor necrosis factor-alpha
t_1/2_: half-life
V_L_: variable light chain domain
3xTg-AD: triple-transgenic mouse model

## References

[1] ALZFORUM | NETWORKING FOR A CURE n.d. http://www.alzforum.org/ (accessed February 10, 2015).

[2] Montoliu-Gaya L, Villegas S. Aβ-Immunotherapeutic strategies: a wide range of approaches for Alzheimer’s disease treatment. Expert Rev Mol Med 2016;18:e13 10.1017/erm.2016.11 27357999

[3] Harrison JR, Owen MJ. Alzheimer’s disease: The amyloid hypothesis on trial. Br J Psychiatry 2016;208:1–3. 10.1192/bjp.bp.115.167569 26729836

[4] Karran E, Mercken M, Strooper B De. The amyloid cascade hypothesis for Alzheimer’s disease: an appraisal for the development of therapeutics. Nat Rev Drug Discov 2011;10:698–712. 10.1038/nrd3505 21852788

[5] Panza F, Frisardi V, Solfrizzi V, Imbimbo BP, Logroscino G, Santamato A, et al Immunotherapy for Alzheimer’s disease: from anti-β-amyloid to tau-based immunization strategies. Immunotherapy 2012;4:213–38. 10.2217/imt.11.170 22339463

[6] Wisniewski T, Goñi F. Immunotherapy for Alzheimer’s disease. Biochem Pharmacol 2014;88:499–507. 10.1016/j.bcp.2013.12.020 24412277 PMC3972315

[7] Fuller JP, Stavenhagen JB, Teeling JL. New roles for Fc receptors in neurodegeneration-the impact on Immunotherapy for Alzheimer’s Disease. Front Neurosci 2014;8:235 10.3389/fnins.2014.00235 25191216 PMC4139653

[8] Güell-Bosch J, Montoliu-Gaya L, Esquerda-Canals G, Villegas S. Aβ immunotherapy for Alzheimer’s disease: where are we? Neurodegener Dis Manag 2016;6:179–81. 10.2217/nmt-2016-0006 27230296

[9] Güell-Bosch J, Esquerda-Canals G, Montoliu-Gaya L, Villegas S. Prospective Therapies for Alzheimer Disease: Biomarkers, Clinical Trials and Preclinical Research. In: Atta-ur-Rahman F, editor. Front. Clin. Drug Res.—CNS Neurol. Disord., Cambridge: 2016, p. Vol.4, 3–80.

[10] ClinicalTrials.gov. US Natl Institutes Heal Bethesda 1988. https://clinicaltrials.gov/ (accessed February 3, 2015).

[11] Salloway S, Sperling R, Fox NC, Blennow K, Klunk W, Raskind M, et al Two phase 3 trials of bapineuzumab in mild-to-moderate Alzheimer’s disease. N Engl J Med 2014;370:322–33. 10.1056/NEJMoa1304839 24450891 PMC4159618

[12] Doody RS, Thomas RG, Farlow M, Iwatsubo T, Vellas B, Joffe S, et al Phase 3 trials of solanezumab for mild-to-moderate Alzheimer’s disease. N Engl J Med 2014;370:311–21. 10.1056/NEJMoa1312889 24450890

[13] Biogen/Eisai Halt Phase 3 Aducanumab Trials | ALZFORUM n.d. https://www.alzforum.org/news/research-news/biogeneisai-halt-phase-3-aducanumab-trials (accessed April 17, 2019).

[14] Holliger P, Hudson PJ. Engineered antibody fragments and the rise of single domains. Nat Biotechnol 2005;23:1126–36. 10.1038/nbt1142 16151406

[15] Monnier P, Vigouroux R, Tassew N. In Vivo Applications of Single Chain Fv (Variable Domain) (scFv) Fragments. Antibodies 2013;2:193–208. 10.3390/antib2020193

[16] Sperling RA, Jack CR, Black SE, Frosch MP, Greenberg SM, Hyman BT, et al Amyloid-related imaging abnormalities in amyloid-modifying therapeutic trials: recommendations from the Alzheimer’s Association Research Roundtable Workgroup. Alzheimers Dement 2011;7:367–85. 10.1016/j.jalz.2011.05.2351 21784348 PMC3693547

[17] Manoutcharian K, Perez-Garmendia R, Gevorkian G. Recombinant Antibody Fragments for Neurodegenerative Diseases. Curr Neuropharmacol 2017;15:779–88. 10.2174/1570159X01666160930121647 27697033 PMC5771054

[18] Huang L, Su X, Federoff H. Single-Chain Fragment Variable Passive Immunotherapies for Neurodegenerative Diseases. Int J Mol Sci 2013;14:19109–27. 10.3390/ijms140919109 24048248 PMC3794823

[19] Frenkel D, Katz O, Solomon B. Immunization against Alzheimer’s beta-amyloid plaques via EFRH phage administration. Proc Natl Acad Sci U S A 2000;97:11455–9. 10.1073/pnas.97.21.11455 11027345 PMC17221

[20] Boado RJ, Lu JZ, Hui EK-W, Pardridge WM. IgG-single chain Fv fusion protein therapeutic for alzheimer’s disease: Expression in CHO cells and pharmacokinetics and brain delivery in the rhesus monkey. Biotechnol Bioeng 2010;105:627–35. 10.1002/bit.22576 19816967 PMC2838425

[21] Wörn A, Plückthun A. Mutual Stabilization of V _L_ and V _H_ in Single-Chain Antibody Fragments, Investigated with Mutants Engineered for Stability ^†^. Biochemistry 1998;37:13120–7. 10.1021/bi980712q 9748318

[22] Ramm K, Gehrig P, Plückthun A. Removal of the conserved disulfide bridges from the scFv fragment of an antibody: effects on folding kinetics and aggregation. J Mol Biol 1999;290:535–46. 10.1006/jmbi.1999.2854 10390351

[23] Wörn A, Plückthun A. Stability engineering of antibody single-chain Fv fragments. J Mol Biol 2001;305:989–1010. 10.1006/jmbi.2000.4265 11162109

[24] Marín-Argany M, Rivera-Hernández G, Martí J, Villegas S. An anti-Aβ (amyloid β) single-chain variable fragment prevents amyloid fibril formation and cytotoxicity by withdrawing Aβ oligomers from the amyloid pathway. Biochem J 2011;437:25–34. 10.1042/BJ20101712 21501114

[25] Montoliu-Gaya L, Murciano-Calles J, Martinez JC, Villegas S. Towards the improvement in stability of an anti-Aβ single-chain variable fragment, scFv-h3D6, as a way to enhance its therapeutic potential. Amyloid 2017;24:167–75. 10.1080/13506129.2017.1348347 28699800

[26] Rivera-Hernández G, Marin-Argany M, Blasco-Moreno B, Bonet J, Oliva B, Villegas S. Elongation of the C-terminal domain of an anti-amyloid β single-chain variable fragment increases its thermodynamic stability and decreases its aggregation tendency. MAbs 2013;5:678–89. 10.4161/mabs.25382 23924802 PMC3851221

[27] Giménez-Llort L, Rivera-Hernández G, Marín-Argany M, Sánchez-Quesada JL, Villegas S. Early intervention in the 3xTg-AD mice with an amyloid β-antibody fragment ameliorates first hallmarks of Alzheimer disease. MAbs 2013;5:665–77. 10.4161/mabs.25424 23884018 PMC3851220

[28] Esquerda-Canals G, Marti J, Rivera-Hernández G, Giménez-Llort L, Villegas S. Loss of deep cerebellar nuclei neurons in the 3xTg-AD mice and protection by an anti-amyloid β antibody fragment. MAbs 2013;5:660–4. 10.4161/mabs.25428 23884149 PMC3851219

[29] Montoliu-Gaya L, Esquerda-Canals G, Bronsoms S, Villegas S. Production of an anti-Aβ antibody fragment in Pichia pastoris and in vitro and in vivo validation of its therapeutic effect. PLoS One 2017;12:e0181480 10.1371/journal.pone.0181480 28771492 PMC5542431

[30] Oddo S, Caccamo A, Shepherd JD, Murphy MP, Golde TE, Kayed R, et al Triple-transgenic model of Alzheimer’s disease with plaques and tangles: intracellular Aβ and synaptic dysfunction. Neuron 2003;39:409–21. 10.1016/S0896-6273(03)00434-3 12895417

[31] Oddo S, Caccamo A, Kitazawa M, Tseng BP, LaFerla FM. Amyloid deposition precedes tangle formation in a triple transgenic model of Alzheimer’s disease. Neurobiol Aging 2003;24:1063–70. 10.1016/j.neurobiolaging.2003.08.012 14643377

[32] Giménez-Llort L, Blázquez G, Cañete T, Johansson B, Oddo S, Tobeña A, et al Modeling behavioral and neuronal symptoms of Alzheimer’s disease in mice: A role for intraneuronal amyloid. Neurosci Biobehav Rev 2007;31:125–47. 10.1016/j.neubiorev.2006.07.007 17055579

[33] Carroll JC, Rosario ER, Kreimer S, Villamagna A, Gentzschein E, Stanczyk FZ, et al Sex differences in β-amyloid accumulation in 3xTg-AD mice: role of neonatal sex steroid hormone exposure. Brain Res 2010;1366:233–45. 10.1016/j.brainres.2010.10.009 20934413 PMC2993873

[34] Montoliu-Gaya L, Güell-Bosch J, Esquerda-Canals G, Roda AR, Serra-Mir G, Lope-Piedrafita S, et al Differential effects of apoE and apoJ mimetic peptides on the action of an anti-Aβ scFv in 3xTg-AD mice. Biochem Pharmacol 2018;155:380–92. 10.1016/j.bcp.2018.07.012 30026023

[35] Franklin KBJ, Paxinos G. The Mouse Brain in Stereotaxic Coordinates. 4th Editio Sydney: Academic Press; 2012.

[36] Güell-Bosch J. Diagnòstic i monitorització de la malaltia d’Alzheimer mitjançant tècniques de ressonància magnètica in vivo en el model 3xTg-AD: Avaluació de l’eficiència del tractament longitudinal amb dos fragments d’anticòs contra el pèptid beta-amiloide Universitat Autònoma de Barcelona, 2017.

[37] Mastrangelo MA, Bowers WJ. Detailed immunohistochemical characterization of temporal and spatial progression of Alzheimer’s disease-related pathologies in male triple-transgenic mice. BMC Neurosci 2008;9:81 10.1186/1471-2202-9-81 18700006 PMC2527610

[38] Esquerda-Canals G, Martí-Clúa J, Roda AR, Villegas S. An Intracellular Amyloid-β/AβPP Epitope Correlates with Neurodegeneration in those Neuronal Populations Early Involved in Alzheimer’s Disease. J Alzheimer’s Dis 2017;59:1079–96. 10.3233/JAD-170218 28697564

[39] García-Cabezas MÁ, John YJ, Barbas H, Zikopoulos B. Distinction of Neurons, Glia and Endothelial Cells in the Cerebral Cortex: An Algorithm Based on Cytological Features. Front Neuroanat 2016;10:107 10.3389/fnana.2016.00107 27847469 PMC5088408

[40] Maier WE, Bartenbach MJ, Brown HW, Tilson HA, Harry GJ. Induction of tumor necrosis factor alpha in cultured glial cells by trimethyltin. Neurochem Int 1997;30:385–92. 9106252 10.1016/s0197-0186(96)00073-3

[41] Iwata A, Iwatsubo T. Disease-modifying therapy for Alzheimer’s disease: Challenges and hopes. Neurol Clin Neurosci 2013;1:49–54. 10.1002/ncn3.20

[42] Benet LZ. Pharmacokinetic parameters: which are necessary to define a drug substance? Eur J Respir Dis Suppl 1984;134:45–61. 6586486

[43] Das P, Howard V, Loosbrock N, Dickson D, Murphy MP, Golde TE. Amyloid-beta immunization effectively reduces amyloid deposition in FcRgamma-/- knock-out mice. J Neurosci 2003;23:8532–8. 13679422 10.1523/JNEUROSCI.23-24-08532.2003 PMC6740360

[44] Kou J, Kim H, Pattanayak A, Song M, Lim J-E, Taguchi H, et al Anti-Amyloid-β Single-Chain Antibody Brain Delivery Via AAV Reduces Amyloid Load But May Increase Cerebral Hemorrhages in an Alzheimer’s Disease Mouse Model. J Alzheimers Dis 2011;27:23–38. 10.3233/JAD-2011-110230 21709371 PMC3560395

[45] Wang Y-J, Pollard A, Zhong J-H, Dong X-Y, Wu X-B, Zhou H-D, et al Intramuscular delivery of a single chain antibody gene reduces brain Abeta burden in a mouse model of Alzheimer’s disease. Neurobiol Aging 2009;30:364–76. 10.1016/j.neurobiolaging.2007.06.013 17686552

[46] Schenk D, Barbour R, Dunn W, Gordon G, Grajeda H, Guido T, et al Immunization with amyloid-beta attenuates Alzheimer-disease-like pathology in the PDAPP mouse. Nature 1999;400:173–7. 10.1038/22124 10408445

[47] Wilcock DM, Rojiani A, Rosenthal A, Levkowitz G, Subbarao S, Alamed J, et al Passive amyloid immunotherapy clears amyloid and transiently activates microglia in a transgenic mouse model of amyloid deposition. J Neurosci 2004;24:6144–51. 10.1523/JNEUROSCI.1090-04.2004 15240806 PMC6729674

[48] Bard F, Cannon C, Barbour R, Burke RL, Games D, Grajeda H, et al Peripherally administered antibodies against amyloid beta-peptide enter the central nervous system and reduce pathology in a mouse model of Alzheimer disease. Nat Med 2000;6:916–9. 10.1038/78682 10932230

[49] DeMattos RB, Bales KR, Cummins DJ, Dodart J-C, Paul SM, Holtzman DM. Peripheral anti-A antibody alters CNS and plasma A clearance and decreases brain A burden in a mouse model of Alzheimer’s disease. Proc Natl Acad Sci 2001;98:8850–5. 10.1073/pnas.151261398 11438712 PMC37524

[50] Robert R, Wark KL. Engineered antibody approaches for Alzheimer’s disease immunotherapy. Arch Biochem Biophys 2012;526:132–8. 10.1016/j.abb.2012.02.022 22475448

[51] Fernandez-Funez P, Zhang Y, Sanchez-Garcia J, de Mena L, Khare S, Golde TE, et al Anti-Aβ single-chain variable fragment antibodies exert synergistic neuroprotective activities in Drosophila models of Alzheimer’s disease. Hum Mol Genet 2015;24:6093–105. 10.1093/hmg/ddv321 26253732 PMC4599669

[52] Watt AD, Crespi GAN, Down RA, Ascher DB, Gunn A, Perez KA, et al Do current therapeutic anti-Aβ antibodies for Alzheimer’s disease engage the target? Acta Neuropathol 2014;127:803–10. 10.1007/s00401-014-1290-2 24803227

[53] Lee G, Cho S, Hoang PM, Kim D, Lee Y, Kil E-J, et al Therapeutic Strategy for the Prevention of Pseudorabies Virus Infection in C57BL/6 Mice by 3D8 scFv with Intrinsic Nuclease Activity. Mol Cells 2015;38:773–80. 10.14348/molcells.2015.0073 26255831 PMC4588720

[54] Lai AY, McLaurin J. Mechanisms of amyloid-Beta Peptide uptake by neurons: the role of lipid rafts and lipid raft-associated proteins. Int J Alzheimers Dis 2010;2011:548380 10.4061/2011/548380 21197446 PMC3010653

[55] Parameswaran N, Patial S. Tumor necrosis factor-α signaling in macrophages. Crit Rev Eukaryot Gene Expr 2010;20:87–103. 21133840 10.1615/critreveukargeneexpr.v20.i2.10 PMC3066460

