# Supplementary figures and images for: Pharmacokinetic parameters and mechanism of action of an efficient anti-Aβ single chain antibody fragment
Source: PLoS One. 2019 May 31;14(5):e0217793. doi: 10.1371/journal.pone.0217793 (PMC6544282; doi:10.1371/journal.pone.0217793)

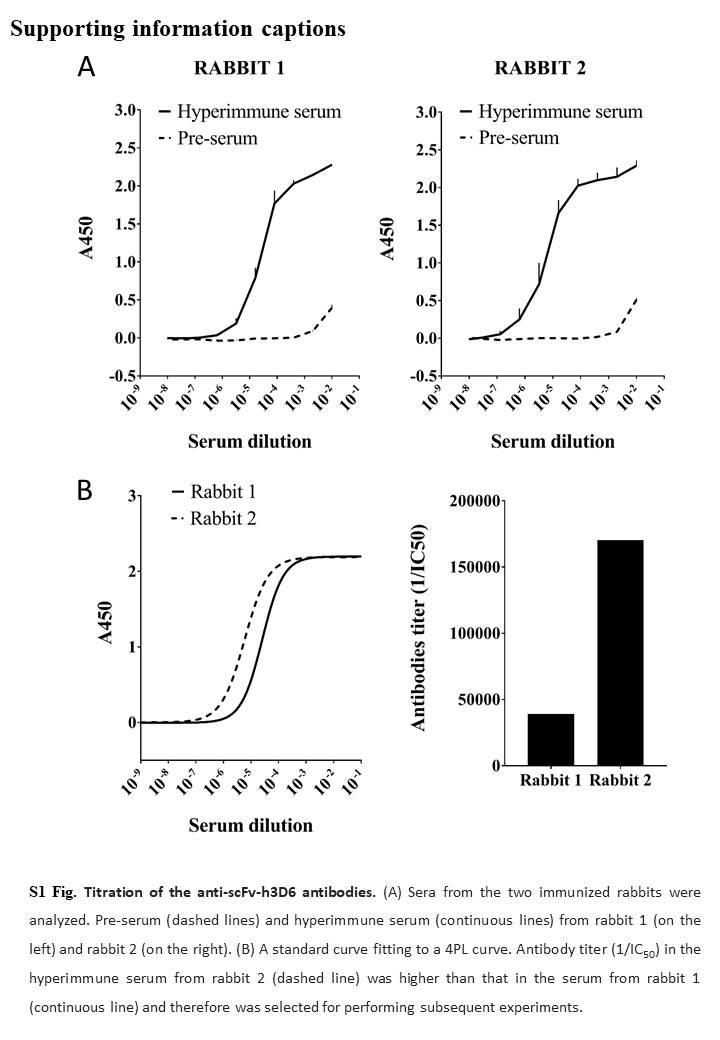

Supplement: S1 Fig — (A) Sera from the two immunized rabbits were analyzed. Pre-serum (dashed lines) and hyperimmune serum (continuous lines) from rabbit 1 (on the left) and rabbit 2 (on the right). (B) A standard curve fitting to a 4PL curve. Antibody titer (1/IC50) in the hyperimmune serum from rabbit 2 (dashed line) was higher than that in the serum from rabbit 1 (continuous line) and therefore was selected for performing subsequent experiments. (TIF) [file pone.0217793.s001.tif]
